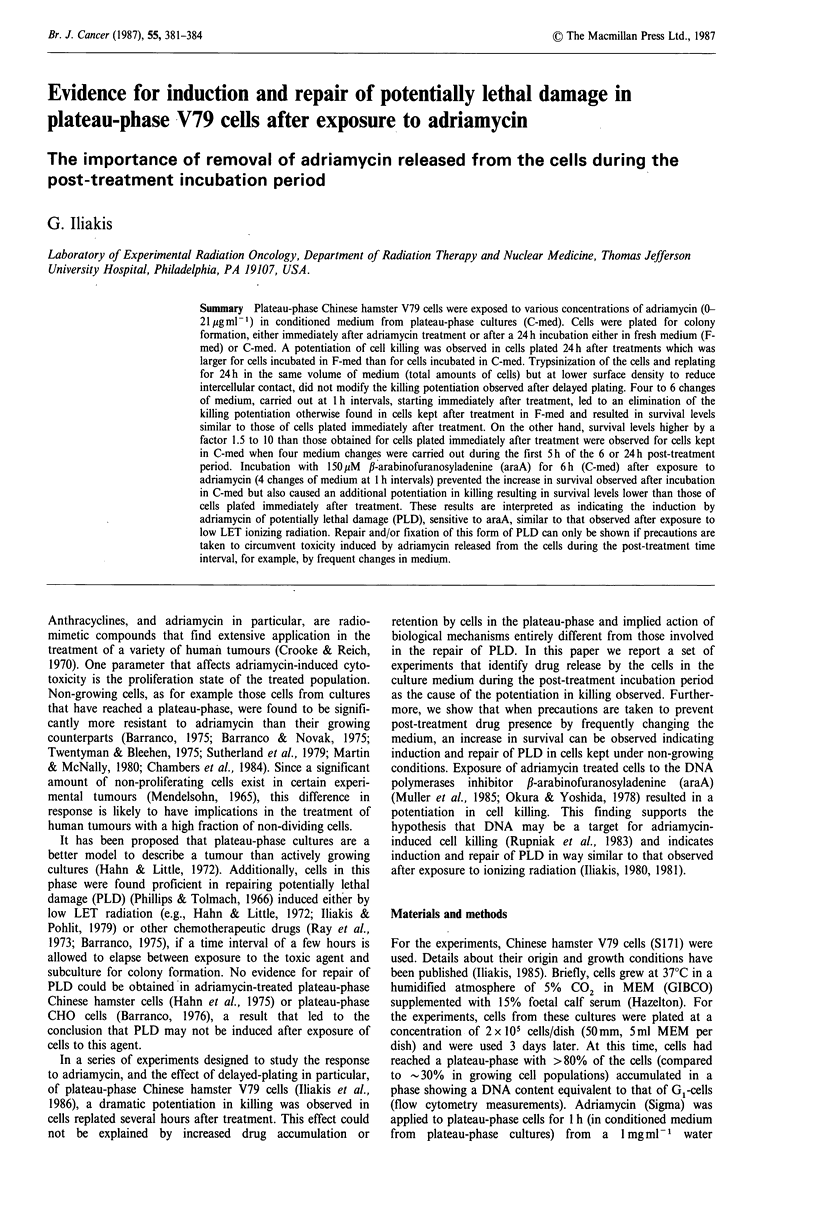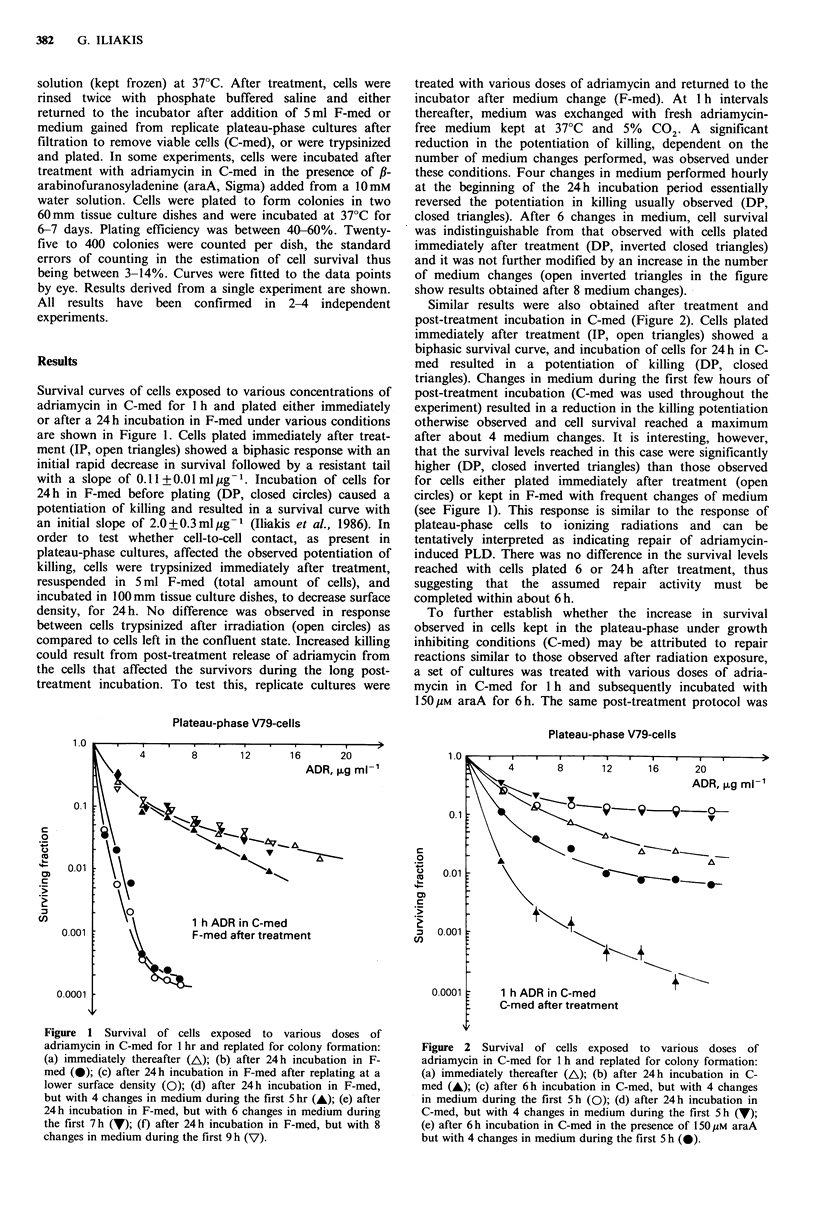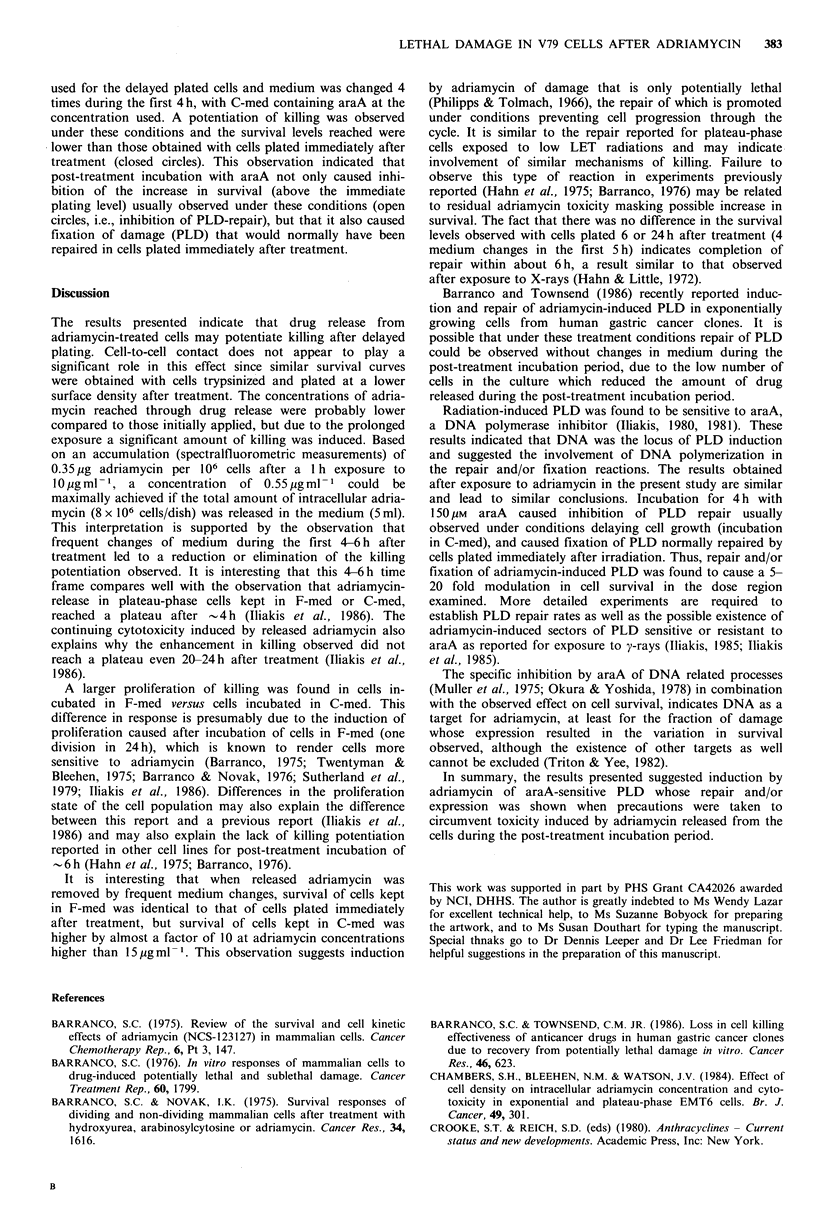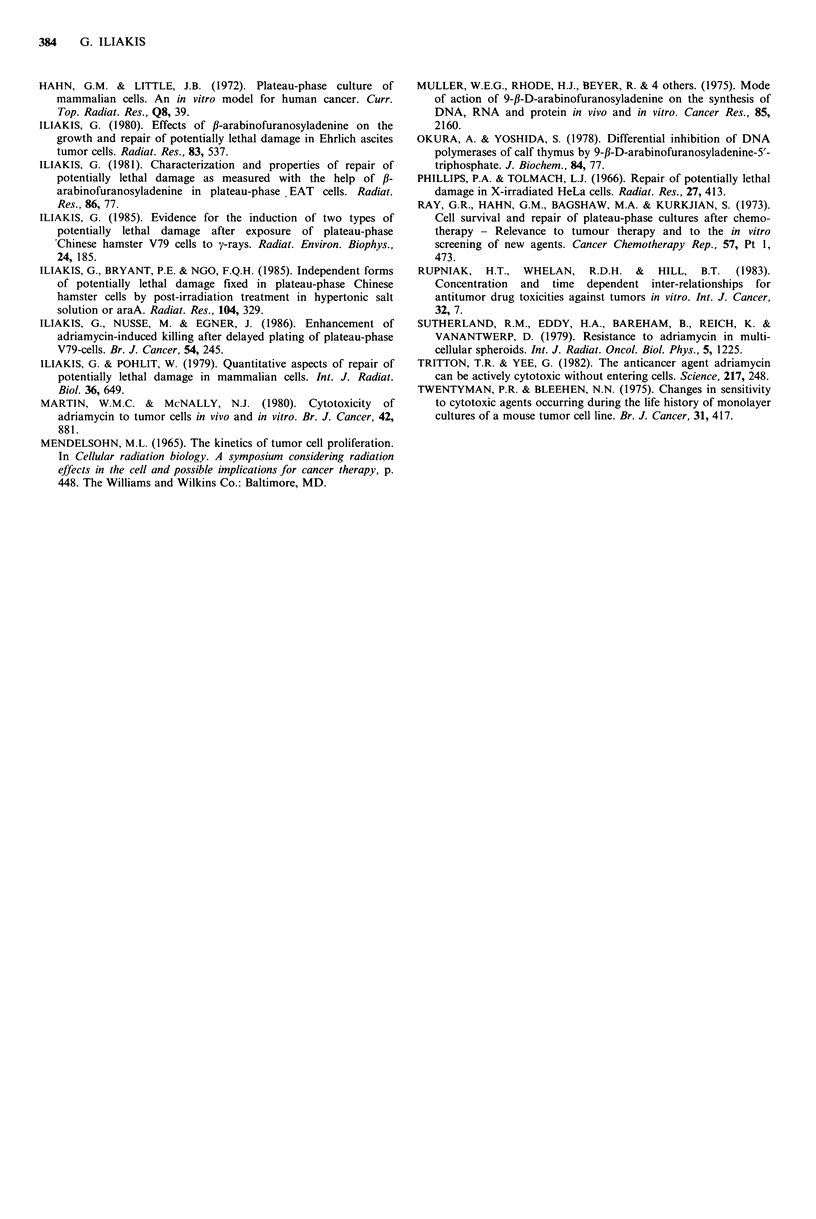# Evidence for induction and repair of potentially lethal damage in plateau-phase V79 cells after exposure to adriamycin. The importance of removal of adriamycin released from the cells during the post-treatment incubation period.

**DOI:** 10.1038/bjc.1987.76

**Published:** 1987-04

**Authors:** G. Iliakis

## Abstract

Plateau-phase Chinese hamster V79 cells were exposed to various concentrations of adriamycin (0-21 micrograms ml-1) in conditioned medium from plateau-phase cultures (C-med). Cells were plated for colony formation, either immediately after adriamycin treatment or after a 24 h incubation either in fresh medium (F-med) or C-med. A potentiation of cell killing was observed in cells plated 24 h after treatments which was larger for cells incubated in F-med than for cells incubated in C-med. Trypsinization of the cells and replating for 24 h in the same volume of medium (total amounts of cells) but at lower surface density to reduce intercellular contact, did not modify the killing potentiation observed after delayed plating. Four to 6 changes of medium, carried out at 1 h intervals, starting immediately after treatment, led to an elimination of the killing potentiation otherwise found in cells kept after treatment in F-med and resulted in survival levels similar to those of cells plated immediately after treatment. On the other hand, survival levels higher by a factor 1.5 to 10 than those obtained for cells plated immediately after treatment were observed for cells kept in C-med when four medium changes were carried out during the first 5 h of the 6 or 24 h post-treatment period. Incubation with 150 microM beta-arabinofuranosyladenine (araA) for 6 h (C-med) after exposure to adriamycin (4 changes of medium at 1 h intervals) prevented the increase in survival observed after incubation in C-med but also caused an additional potentiation in killing resulting in survival levels lower than those of cells plated immediately after treatment. These results are interpreted as indicating the induction by adriamycin of potentially lethal damage (PLD), sensitive to araA, similar to that observed after exposure to low LET ionizing radiation. Repair and/or fixation of this form of PLD can only be shown if precautions are taken to circumvent toxicity induced by adriamycin released from the cells during the post-treatment time interval, for example, by frequent changes in medium.


Br. J. Cancer (1987), 55, 381–384 © The Macmillan Press Ltd., 1987

### Evidence for induction and repair of potentially lethal damage in plateau-phase V79 cells after exposure to adriamycin

### The importance of removal of adriamycin released from the cells during the post-treatment incubation period

G. Iliakis

*Laboratory of Experimental Radiation Oncology, Department of Radiation Therapy and Nuclear Medicine, Thomas Jefferson University Hospital, Philadelphia, PA 19107, USA.*

**Summary** Plateau-phase Chinese hamster V79 cells were exposed to various concentrations of adriamycin (0–21 $\mu g\,ml^{-1}$) in conditioned medium from plateau-phase cultures (C-med). Cells were plated for colony formation, either immediately after adriamycin treatment or after a 24 h incubation either in fresh medium (F-med) or C-med. A potentiation of cell killing was observed in cells plated 24 h after treatments which was larger for cells incubated in F-med than for cells incubated in C-med. Trypsinization of the cells and replating for 24 h in the same volume of medium (total amounts of cells) but at lower surface density to reduce intercellular contact, did not modify the killing potentiation observed after delayed plating. Four to 6 changes of medium, carried out at 1 h intervals, starting immediately after treatment, led to an elimination of the killing potentiation otherwise found in cells kept after treatment in F-med and resulted in survival levels similar to those of cells plated immediately after treatment. On the other hand, survival levels higher by a factor 1.5 to 10 than those obtained for cells plated immediately after treatment were observed for cells kept in C-med when four medium changes were carried out during the first 5 h of the 6 or 24 h post-treatment period. Incubation with 150 $\mu M$ $\beta$-arabinofuranosyladenine (araA) for 6 h (C-med) after exposure to adriamycin (4 changes of medium at 1 h intervals) prevented the increase in survival observed after incubation in C-med but also caused an additional potentiation in killing resulting in survival levels lower than those of cells plated immediately after treatment. These results are interpreted as indicating the induction by adriamycin of potentially lethal damage (PLD), sensitive to araA, similar to that observed after exposure to low LET ionizing radiation. Repair and/or fixation of this form of PLD can only be shown if precautions are taken to circumvent toxicity induced by adriamycin released from the cells during the post-treatment time interval, for example, by frequent changes in medium.

Anthracyclines, and adriamycin in particular, are radiomimetic compounds that find extensive application in the treatment of a variety of human tumours (Crooke & Reich, 1970). One parameter that affects adriamycin-induced cytotoxicity is the proliferation state of the treated population. Non-growing cells, as for example those cells from cultures that have reached a plateau-phase, were found to be significantly more resistant to adriamycin than their growing counterparts (Barranco, 1975; Barranco & Novak, 1975; Twentyman & Bleehen, 1975; Sutherland *et al.*, 1979; Martin & McNally, 1980; Chambers *et al.*, 1984). Since a significant amount of non-proliferating cells exist in certain experimental tumours (Mendelsohn, 1965), this difference in response is likely to have implications in the treatment of human tumours with a high fraction of non-dividing cells.

It has been proposed that plateau-phase cultures are a better model to describe a tumour than actively growing cultures (Hahn & Little, 1972). Additionally, cells in this phase were found proficient in repairing potentially lethal damage (PLD) (Phillips & Tolmach, 1966) induced either by low LET radiation (e.g., Hahn & Little, 1972; Iliakis & Pohlit, 1979) or other chemotherapeutic drugs (Ray *et al.*, 1973; Barranco, 1975), if a time interval of a few hours is allowed to elapse between exposure to the toxic agent and subculture for colony formation. No evidence for repair of PLD could be obtained in adriamycin-treated plateau-phase Chinese hamster cells (Hahn *et al.*, 1975) or plateau-phase CHO cells (Barranco, 1976), a result that led to the conclusion that PLD may not be induced after exposure of cells to this agent.

In a series of experiments designed to study the response to adriamycin, and the effect of delayed-plating in particular, of plateau-phase Chinese hamster V79 cells (Iliakis *et al.*, 1986), a dramatic potentiation in killing was observed in cells replated several hours after treatment. This effect could not be explained by increased drug accumulation or retention by cells in the plateau-phase and implied action of biological mechanisms entirely different from those involved in the repair of PLD. In this paper we report a set of experiments that identify drug release by the cells in the culture medium during the post-treatment incubation period as the cause of the potentiation in killing observed. Furthermore, we show that when precautions are taken to prevent post-treatment drug presence by frequently changing the medium, an increase in survival can be observed indicating induction and repair of PLD in cells kept under non-growing conditions. Exposure of adriamycin treated cells to the DNA polymerases inhibitor $\beta$-arabinofuranosyladenine (araA) (Muller *et al.*, 1985; Okura & Yoshida, 1978) resulted in a potentiation in cell killing. This finding supports the hypothesis that DNA may be a target for adriamycin-induced cell killing (Rupniak *et al.*, 1983) and indicates induction and repair of PLD in way similar to that observed after exposure to ionizing radiation (Iliakis, 1980, 1981).

### Materials and methods

For the experiments, Chinese hamster V79 cells (S171) were used. Details about their origin and growth conditions have been published (Iliakis, 1985). Briefly, cells grew at 37°C in a humidified atmosphere of 5% $CO_2$ in MEM (GIBCO) supplemented with 15% foetal calf serum (Hazelton). For the experiments, cells from these cultures were plated at a concentration of $2\times10^5$ cells/dish (50 mm, 5 ml MEM per dish) and were used 3 days later. At this time, cells had reached a plateau-phase with >80% of the cells (compared to ~30% in growing cell populations) accumulated in a phase showing a DNA content equivalent to that of $G_1$-cells (flow cytometry measurements). Adriamycin (Sigma) was applied to plateau-phase cells for 1 h (in conditioned medium from plateau-phase cultures) from a 1 $mg\,ml^{-1}$ water solution (kept frozen) at 37°C. After treatment, cells were rinsed twice with phosphate buffered saline and either returned to the incubator after addition of 5 ml F-med or medium gained from replicate plateau-phase cultures after filtration to remove viable cells (C-med), or were trypsinized and plated. In some experiments, cells were incubated after treatment with adriamycin in C-med in the presence of $\beta$-arabinofuranosyladenine (araA, Sigma) added from a 10 mM water solution. Cells were plated to form colonies in two 60 mm tissue culture dishes and were incubated at 37°C for 6–7 days. Plating efficiency was between 40–60%. Twenty-five to 400 colonies were counted per dish, the standard errors of counting in the estimation of cell survival thus being between 3–14%. Curves were fitted to the data points by eye. Results derived from a single experiment are shown. All results have been confirmed in 2–4 independent experiments.

### Results

Survival curves of cells exposed to various concentrations of adriamycin in C-med for 1 h and plated either immediately or after a 24 h incubation in F-med under various conditions are shown in Figure 1. Cells plated immediately after treatment (IP, open triangles) showed a biphasic response with an initial rapid decrease in survival followed by a resistant tail with a slope of $0.11 \pm 0.01$ ml $\mu g^{-1}$. Incubation of cells for 24 h in F-med before plating (DP, closed circles) caused a potentiation of killing and resulted in a survival curve with an initial slope of $2.0 \pm 0.3$ ml $\mu g^{-1}$ (Iliakis *et al.*, 1986). In order to test whether cell-to-cell contact, as present in plateau-phase cultures, affected the observed potentiation of killing, cells were trypsinized immediately after treatment, resuspended in 5 ml F-med (total amount of cells), and incubated in 100 mm tissue culture dishes, to decrease surface density, for 24 h. No difference was observed in response between cells trypsinized after irradiation (open circles) as compared to cells left in the confluent state. Increased killing could result from post-treatment release of adriamycin from the cells that affected the survivors during the long post-treatment incubation. To test this, replicate cultures were treated with various doses of adriamycin and returned to the incubator after medium change (F-med). At 1 h intervals thereafter, medium was exchanged with fresh adriamycin-free medium kept at 37°C and 5% $CO_2$. A significant reduction in the potentiation of killing, dependent on the number of medium changes performed, was observed under these conditions. Four changes in medium performed hourly at the beginning of the 24 h incubation period essentially reversed the potentiation in killing usually observed (DP, closed triangles). After 6 changes in medium, cell survival was indistinguishable from that observed with cells plated immediately after treatment (DP, inverted closed triangles) and it was not further modified by an increase in the number of medium changes (open inverted triangles in the figure show results obtained after 8 medium changes).

Similar results were also obtained after treatment and post-treatment incubation in C-med (Figure 2). Cells plated immediately after treatment (IP, open triangles) showed a biphasic survival curve, and incubation of cells for 24 h in C-med resulted in a potentiation of killing (DP, closed triangles). Changes in medium during the first few hours of post-treatment incubation (C-med was used throughout the experiment) resulted in a reduction in the killing potentiation otherwise observed and cell survival reached a maximum after about 4 medium changes. It is interesting, however, that the survival levels reached in this case were significantly higher (DP, closed inverted triangles) than those observed for cells either plated immediately after treatment (open circles) or kept in F-med with frequent changes of medium (see Figure 1). This response is similar to the response of plateau-phase cells to ionizing radiations and can be tentatively interpreted as indicating repair of adriamycin-induced PLD. There was no difference in the survival levels reached with cells plated 6 or 24 h after treatment, thus suggesting that the assumed repair activity must be completed within about 6 h.

To further establish whether the increase in survival observed in cells kept in the plateau-phase under growth inhibiting conditions (C-med) may be attributed to repair reactions similar to those observed after radiation exposure, a set of cultures was treated with various doses of adriamycin in C-med for 1 h and subsequently incubated with 150 $\mu$M araA for 6 h. The same post-treatment protocol was

**Figure 1** Survival of cells exposed to various doses of adriamycin in C-med for 1 hr and replated for colony formation: (a) immediately thereafter (△); (b) after 24 h incubation in F-med (●); (c) after 24 h incubation in F-med after replating at a lower surface density (○); (d) after 24 h incubation in F-med, but with 4 changes in medium during the first 5 hr (▲); (e) after 24 h incubation in F-med, but with 6 changes in medium during the first 7 h (▼); (f) after 24 h incubation in F-med, but with 8 changes in medium during the first 9 h (▽).

**Figure 2** Survival of cells exposed to various doses of adriamycin in C-med for 1 h and replated for colony formation: (a) immediately thereafter (△); (b) after 24 h incubation in C-med (▲); (c) after 6 h incubation in C-med, but with 4 changes in medium during the first 5 h (○); (d) after 24 h incubation in C-med, but with 4 changes in medium during the first 5 h (▼); (e) after 6 h incubation in C-med in the presence of 150 $\mu$M araA but with 4 changes in medium during the first 5 h (●).

used for the delayed plated cells and medium was changed 4 times during the first 4 h, with C-med containing araA at the concentration used. A potentiation of killing was observed under these conditions and the survival levels reached were lower than those obtained with cells plated immediately after treatment (closed circles). This observation indicated that post-treatment incubation with araA not only caused inhibition of the increase in survival (above the immediate plating level) usually observed under these conditions (open circles, i.e., inhibition of PLD-repair), but that it also caused fixation of damage (PLD) that would normally have been repaired in cells plated immediately after treatment.

### Discussion

The results presented indicate that drug release from adriamycin-treated cells may potentiate killing after delayed plating. Cell-to-cell contact does not appear to play a significant role in this effect since similar survival curves were obtained with cells trypsinized and plated at a lower surface density after treatment. The concentrations of adriamycin reached through drug release were probably lower compared to those initially applied, but due to the prolonged exposure a significant amount of killing was induced. Based on an accumulation (spectralfluorometric measurements) of 0.35 $\mu$g adriamycin per $10^6$ cells after a 1 h exposure to 10 $\mu g\,ml^{-1}$, a concentration of 0.55 $\mu g\,ml^{-1}$ could be maximally achieved if the total amount of intracellular adriamycin ($8 \times 10^6$ cells/dish) was released in the medium (5 ml). This interpretation is supported by the observation that frequent changes of medium during the first 4–6 h after treatment led to a reduction or elimination of the killing potentiation observed. It is interesting that this 4–6 h time frame compares well with the observation that adriamycin-release in plateau-phase cells kept in F-med or C-med, reached a plateau after ~4 h (Iliakis *et al.*, 1986). The continuing cytotoxicity induced by released adriamycin also explains why the enhancement in killing observed did not reach a plateau even 20–24 h after treatment (Iliakis *et al.*, 1986).

A larger proliferation of killing was found in cells incubated in F-med *versus* cells incubated in C-med. This difference in response is presumably due to the induction of proliferation caused after incubation of cells in F-med (one division in 24 h), which is known to render cells more sensitive to adriamycin (Barranco, 1975; Twentyman & Bleehen, 1975; Barranco & Novak, 1976; Sutherland *et al.*, 1979; Iliakis *et al.*, 1986). Differences in the proliferation state of the cell population may also explain the difference between this report and a previous report (Iliakis *et al.*, 1986) and may also explain the lack of killing potentiation reported in other cell lines for post-treatment incubation of ~6 h (Hahn *et al.*, 1975; Barranco, 1976).

It is interesting that when released adriamycin was removed by frequent medium changes, survival of cells kept in F-med was identical to that of cells plated immediately after treatment, but survival of cells kept in C-med was higher by almost a factor of 10 at adriamycin concentrations higher than 15 $\mu g\,ml^{-1}$. This observation suggests induction by adriamycin of damage that is only potentially lethal (Philipps & Tolmach, 1966), the repair of which is promoted under conditions preventing cell progression through the cycle. It is similar to the repair reported for plateau-phase cells exposed to low LET radiations and may indicate involvement of similar mechanisms of killing. Failure to observe this type of reaction in experiments previously reported (Hahn *et al.*, 1975; Barranco, 1976) may be related to residual adriamycin toxicity masking possible increase in survival. The fact that there was no difference in the survival levels observed with cells plated 6 or 24 h after treatment (4 medium changes in the first 5 h) indicates completion of repair within about 6 h, a result similar to that observed after exposure to X-rays (Hahn & Little, 1972).

Barranco and Townsend (1986) recently reported induction and repair of adriamycin-induced PLD in exponentially growing cells from human gastric cancer clones. It is possible that under these treatment conditions repair of PLD could be observed without changes in medium during the post-treatment incubation period, due to the low number of cells in the culture which reduced the amount of drug released during the post-treatment incubation period.

Radiation-induced PLD was found to be sensitive to araA, a DNA polymerase inhibitor (Iliakis, 1980, 1981). These results indicated that DNA was the locus of PLD induction and suggested the involvement of DNA polymerization in the repair and/or fixation reactions. The results obtained after exposure to adriamycin in the present study are similar and lead to similar conclusions. Incubation for 4 h with 150 $\mu$M araA caused inhibition of PLD repair usually observed under conditions delaying cell growth (incubation in C-med), and caused fixation of PLD normally repaired by cells plated immediately after irradiation. Thus, repair and/or fixation of adriamycin-induced PLD was found to cause a 5–20 fold modulation in cell survival in the dose region examined. More detailed experiments are required to establish PLD repair rates as well as the possible existence of adriamycin-induced sectors of PLD sensitive or resistant to araA as reported for exposure to $\gamma$-rays (Iliakis, 1985; Iliakis *et al.*, 1985).

The specific inhibition by araA of DNA related processes (Muller *et al.*, 1975; Okura & Yoshida, 1978) in combination with the observed effect on cell survival, indicates DNA as a target for adriamycin, at least for the fraction of damage whose expression resulted in the variation in survival observed, although the existence of other targets as well cannot be excluded (Triton & Yee, 1982).

In summary, the results presented suggested induction by adriamycin of araA-sensitive PLD whose repair and/or expression was shown when precautions were taken to circumvent toxicity induced by adriamycin released from the cells during the post-treatment incubation period.

This work was supported in part by PHS Grant CA42026 awarded by NCI, DHHS. The author is greatly indebted to Ms Wendy Lazar for excellent technical help, to Ms Suzanne Bobyock for preparing the artwork, and to Ms Susan Douthart for typing the manuscript. Special thnaks go to Dr Dennis Leeper and Dr Lee Friedman for helpful suggestions in the preparation of this manuscript.

### References

BARRANCO, S.C. (1975). Review of the survival and cell kinetic effects of adriamycin (NCS-123127) in mammalian cells. *Cancer Chemotherapy Rep.*, **6**, Pt 3, 147.

BARRANCO, S.C. (1976). *In vitro* responses of mammalian cells to drug-induced potentially lethal and sublethal damage. *Cancer Treatment Rep.*, **60**, 1799.

BARRANCO, S.C. & NOVAK, I.K. (1975). Survival responses of dividing and non-dividing mammalian cells after treatment with hydroxyurea, arabinosylcytosine or adriamycin. *Cancer Res.*, **34**, 1616.

BARRANCO, S.C. & TOWNSEND, C.M. JR. (1986). Loss in cell killing effectiveness of anticancer drugs in human gastric cancer clones due to recovery from potentially lethal damage *in vitro*. *Cancer Res.*, **46**, 623.

CHAMBERS, S.H., BLEEHEN, N.M. & WATSON, J.V. (1984). Effect of cell density on intracellular adriamycin concentration and cytotoxicity in exponential and plateau-phase EMT6 cells. *Br. J. Cancer*, **49**, 301.

CROOKE, S.T. & REICH, S.D. (eds) (1980). *Anthracyclines – Current status and new developments*. Academic Press, Inc: New York.

HAHN, G.M. & LITTLE, J.B. (1972). Plateau-phase culture of mammalian cells. An *in vitro* model for human cancer. *Curr. Top. Radiat. Res.*, **Q8,** 39.

ILIAKIS, G. (1980). Effects of β-arabinofuranosyladenine on the growth and repair of potentially lethal damage in Ehrlich ascites tumor cells. *Radiat. Res.*, **83,** 537.

ILIAKIS, G. (1981). Characterization and properties of repair of potentially lethal damage as measured with the help of β-arabinofuranosyladenine in plateau-phase EAT cells. *Radiat. Res.*, **86,** 77.

ILIAKIS, G. (1985). Evidence for the induction of two types of potentially lethal damage after exposure of plateau-phase Chinese hamster V79 cells to γ-rays. *Radiat. Environ. Biophys.*, **24,** 185.

ILIAKIS, G., BRYANT, P.E. & NGO, F.Q.H. (1985). Independent forms of potentially lethal damage fixed in plateau-phase Chinese hamster cells by post-irradiation treatment in hypertonic salt solution or araA. *Radiat. Res.*, **104,** 329.

ILIAKIS, G., NUSSE, M. & EGNER, J. (1986). Enhancement of adriamycin-induced killing after delayed plating of plateau-phase V79-cells. *Br. J. Cancer*, **54,** 245.

ILIAKIS, G. & POHLIT, W. (1979). Quantitative aspects of repair of potentially lethal damage in mammalian cells. *Int. J. Radiat. Biol.* **36,** 649.

MARTIN, W.M.C. & McNALLY, N.J. (1980). Cytotoxicity of adriamycin to tumor cells *in vivo* and *in vitro*. *Br. J. Cancer*, **42,** 881.

MENDELSOHN, M.L. (1965). The kinetics of tumor cell proliferation. In *Cellular radiation biology. A symposium considering radiation effects in the cell and possible implications for cancer therapy*, p. 448. The Williams and Wilkins Co.: Baltimore, MD.

MULLER, W.E.G., RHODE, H.J., BEYER, R. & 4 others. (1975). Mode of action of 9-β-D-arabinofuranosyladenine on the synthesis of DNA, RNA and protein *in vivo* and *in vitro*. *Cancer Res.*, **85,** 2160.

OKURA, A. & YOSHIDA, S. (1978). Differential inhibition of DNA polymerases of calf thymus by 9-β-D-arabinofuranosyladenine-5′-triphosphate. *J. Biochem.*, **84,** 77.

PHILLIPS, P.A. & TOLMACH, L.J. (1966). Repair of potentially lethal damage in X-irradiated HeLa cells. *Radiat. Res.*, **27,** 413.

RAY, G.R., HAHN, G.M., BAGSHAW, M.A. & KURKJIAN, S. (1973). Cell survival and repair of plateau-phase cultures after chemotherapy – Relevance to tumour therapy and to the *in vitro* screening of new agents. *Cancer Chemotherapy Rep.*, **57,** Pt 1, 473.

RUPNIAK, H.T., WHELAN, R.D.H. & HILL, B.T. (1983). Concentration and time dependent inter-relationships for antitumor drug toxicities against tumors *in vitro*. *Int. J. Cancer*, **32,** 7.

SUTHERLAND, R.M., EDDY, H.A., BAREHAM, B., REICH, K. & VANANTWERP, D. (1979). Resistance to adriamycin in multicellular spheroids. *Int. J. Radiat. Oncol. Biol. Phys.*, **5,** 1225.

TRITTON, T.R. & YEE, G. (1982). The anticancer agent adriamycin can be actively cytotoxic without entering cells. *Science*, **217,** 248.

TWENTYMAN, P.R. & BLEEHEN, N.N. (1975). Changes in sensitivity to cytotoxic agents occurring during the life history of monolayer cultures of a mouse tumor cell line. *Br. J. Cancer*, **31,** 417.